# Death of Rudolphi

**Published:** 1833-01

**Authors:** 


### OBITUARY.

Death of Rudolphi. M. Rudolphi died at Berlin, on the 29th November, in the sixty-third year of his age. He was highly distinguished as a practical physician, an ingenious experimental physiologist, and for many important contributions to natural history. His life was spent in the diligent cultivation of every species of knowledge which could secure him a perfect acquaintance with his profession, and his reputation as a man of first-rate talent was acknowledged and duly appreciated throughout the whole of Europe.

